# Effect of music intervention on subjective scores, heart rate variability, and prefrontal hemodynamics in patients with chronic pain

**DOI:** 10.3389/fnhum.2022.1057290

**Published:** 2022-11-17

**Authors:** Jiahao Du, Ping Shi, Fanfu Fang, Hongliu Yu

**Affiliations:** ^1^Institute of Rehabilitation Engineering and Technology, University of Shanghai for Science and Technology, Shanghai, China; ^2^Department of Rehabilitation Medicine, Changhai Hospital, Naval Medical University, Shanghai, China

**Keywords:** chronic pain, functional near-infrared spectroscopy, heart rate variability, music intervention, prefrontal hemodynamics

## Abstract

**Introduction:**

Music interventions have been proposed in recent years as a treatment for chronic pain. However, the mechanisms by which music relieves pain are unclear, and the effects of music intervention on physiological indicators in patients with chronic pain remain to be explored. This study aimed to explore whether a music intervention would have effects on subjective pain ratings, heart rate variability, and functional connectivity of the cerebral cortex in patients with chronic pain.

**Methods:**

A randomized controlled study was conducted on 37 pain patients aged 18–65 years, with the control group receiving usual care, and the intervention group receiving music intervention (8–150 Hz, 50–70 dB) for 30 min before bedtime for 7 days on top of usual care. Pain visual analog scale and heart rate variability were used as subjective and objective physiological indices before and after the music intervention, respectively. Changes in oxyhemoglobin and deoxyhemoglobin concentrations in the cerebral cortex were measured by functional near-infrared spectroscopy, and whole-brain correlation analysis was used to quantify the connectivity of prefrontal brain regions associated with the pain response.

**Results:**

Results showed that patients with chronic pain in the intervention group had significantly lower visual assessment scale scores, as well as significantly lower overall voluntary mobility during pain episodes, resulting in relatively higher vagal innervation compared to the control group. In addition, connections between the bilateral dorsolateral prefrontal cortex (BA9, BA46) and frontal areas (BA10) were significantly higher in the intervention group.

**Discussion:**

This study demonstrates the effectiveness of the combined application of music interventions with usual care in reducing pain levels in patients with chronic pain and provides insight into the pathological mechanisms of music interventions for analgesia, providing direction for new baseline indicators for quantitative clinical assessment of pain. The study was registered in the Chinese Clinical Trial Registry (No. ChiCTR2100052993).

**Clinical trial registration:**

[https://www.chictr.org.cn/showproj.aspx?proj=136268], identifier [ChiCTR2100052993].

## Introduction

Episodes of chronic pain are generally considered particularly distressing, and chronic pain without respite can even cause depression and anxiety that interfere with the patient’s normal life. Over the past 20 years, opioids have been the primary treatment choice for chronic pain, but with poor long-term outcomes and the potential for opioid dependence (Manhapra, 2022). The high prevalence, burden, and refractoriness of chronic pain and the opioid dependence crisis have drawn the attention of scholars to complementary or alternative therapies (EBioMedicine, 2016).

Among various non-pharmacological interventions, music interventions are widely used for chronic pain relief due to their advantages such as inexpensive, non-discriminatory, and non-adverse effects (Sihvonen et al., 2017). A growing body of evidence supports the use of music interventions for pain management (Martin-Saavedra et al., 2018; Howlin and Rooney, 2021; Hsu et al., 2022). Hsu et al. (2022) implemented a music intervention in 66 elderly patients with osteoarthritis, and the visual analog scales (VAS) scores were significantly lower in the intervention group than in the control group. Some researchers have found that music intervention may reduce pain levels in patients with chronic pain, and have significant positive effects on physical comfort and relaxation (Howlin and Rooney, 2021; Ting et al., 2022).

The clinical use of music interventions in pain medicine is promising. Psychology argues that music interventions can improve motivation, elevate mood, and relieve pain by intentionally distracting people from unpleasant sensations and reducing the perception of pain or anxiety (Kristjánsdóttir and Kristjánsdóttir, 2011). On the other hand, neural mechanism theories suggest that the melody and rhythm of music influence autonomic nervous system regulation and pain perception by influencing the limbic system and hypothalamus to reduce the secretion of catecholamines (Loewy, 2022). Musical melodies of 8–13 Hz stimulate alpha brain waves to relax consciousness (Han et al., 2022). Also, music interventions promote high endorphin secretion and lower blood pressure, heart rate, respiratory rate, oxygen consumption, and plasma lactate levels, thus contributing to pain relief (Wang et al., 2020c). A recent study (Zhou et al., 2022) revealed the mechanism by which music induces analgesia through corticothalamic circuits through experiments on mice. The mechanisms by which music interventions are effective for chronic pain are still being explored.

Exploring the effectiveness of chronic pain interventions through changes in objective physiological indicators has gradually become a hot topic in pain assessment (Ling et al., 2014; Jess et al., 2016; Boissoneault et al., 2019). In clinical practice, patients with chronic pain often exhibit signs of the autonomic nervous system, such as fatigue, sleep disturbances, and decreased appetite, which affect pain VAS (Wang et al., 2020a) and the level of heart rate variability (HRV) (Forte et al., 2022). It was found that during fibromyalgia episodes, the low-frequency (sympathetic) power spectrum increases, and the high-frequency (vagus) decreases, with a corresponding increase in pain VAS (Reneau, 2020). Functional neuroimaging studies have shown pain-related activation in several cortical areas during chronic pain, including the prefrontal, cingulate, insula, and primary and secondary somatosensory cortices (Morton et al., 2016). The Prefrontal is primarily responsible for the perception of and response to pain (Wang et al., 2020b). Changes in prefrontal cortex blood flow concentration are associated with chronic pain episodes captured by functional near-infrared spectroscopy (fNIRS) (Öztürk et al., 2021). Furthermore, it was observed that the functional connectivity in the prefrontal cortex decreases during pain episodes, while network connectivity increases after the local intervention, which was associated with individual pain relief (Kim et al., 2020). Functional connectivity between the medial prefrontal cortex and frontopolar cortex has also been shown to be improved in patients with trigeminal neuralgia (Tsai et al., 2018). However, few studies have explored the effectiveness of chronic pain interventions through a combination of VAS, heart rate variability, and brain network functional connectivity.

To investigate the effectiveness of music intervention for chronic pain, we compared the changes in VAS, heart rate (HR), and HRV of patients before and after music intervention. We are also interested in the impact of music intervention on the functional connectivity of the prefrontal cortex when assessing pain in patients suffering from chronic pain. We expect the music intervention to promote better functional connectivity between the dorsolateral prefrontal cortex and frontopolar area during pain assessment in patients with chronic pain, while decreasing pain scores and autonomous activity in pain patients.

## Methods

The study was registered before patient recruitment and was conducted by the Department of Pain, Huadong Hospital Affiliated to Fudan University, Shanghai, China, from December 15, 2021, to February 28, 2022. This study has completed ethical approval (2021K104-F221) and Chinese clinical trial registration (ChiCTR2100052993). After receiving verbal and written study information, an approved consent form was signed by the patient in case of study participation.

### Participants

Eligible participants ranged in age from 18 to 65 years with chronic pain. Additionally, cognitive impairments, antipsychotic or antidepressant drugs in the past three months, insensitivity to the audio used in this experiment, being in psychotherapy, and other serious illnesses were identified as exclusion criteria.

Sample size estimation was based on the effect and standard variation found in the systematic review (Hsu et al., 2022), and was conducted with G*power 3.1 (Faul et al., 2009) setting α = 0.05 and β = 0.20. It was found that 17 participants were needed in each group. We included 19 patients in each group considering attrition.

Participants were randomly assigned to the following two groups in a 1:1 allocation ratio. An independent statistician randomized blocks of random sizes, hiding the task from clinicians and patients.

### Procedures

#### Experimental paradigm

A pretest-posttest design was used, with one music intervention group and one control group. A repeated-measures design was designed, in which each patient’s pain VAS and electrocardiogram (ECG) recordings were measured before and after the intervention. The measurements required participants to refrain from alcohol for 24 h and caffeinated beverages and nicotine for 4 h prior to this examination, as these substances have known effects on nociception and heart rate variability.

In addition, we immediately monitored the cerebral hemodynamics of the patients through fNIRS after the intervention, and the patients in the intervention group always wore headphones to listen to music.

#### Music intervention

Music with a frequency of 8–13 Hz can stimulate alpha brainwaves to aid mental relaxation (Huang et al., 2016). Low-frequency somatic vibration music with frequencies of 16–150 Hz can promote sleep and stabilize mood (Kühlmann et al., 2018). Considering that 60–85 dB is the optimal sound threshold range for the human ear (White, 2000), the researchers suggested that music interventions at a volume of 55–70 dB (Standley, 2002), 10 dB higher than ambient noise, would be appropriate.

The participants in the control group underwent 7 days of usual care (appropriate exercise directed by a clinician), while participants in the intervention group listened to specific music (8–150 Hz, 50–70 dB) for 30 min each day before bedtime for 7 days on top of their usual care.

#### Measurements

##### Pain visual analog scale

The Pain VAS is a pain intensity assessment tool that changes from a score of 1 to 10 to indicate pain levels ranging from pain-free to extreme pain.

##### Electrocardiogram acquisition

Participants were asked to sit upright in a chair and keep their bodies stable for 5 min of ECG signal acquisition, and to maintain natural respiration to avoid motion artifacts. In this study, the Power-Lab/16sp system (Castle Hill, AD Instruments, Australia) was used to acquire and amplify ECG signals at a sampling rate of 1 kHz. The ECG signals were then filtered through a 45 Hz low-pass filter and a 1 Hz high-pass filter. The RR interval sequence of the ECG signals was calculated, and the time-domain parameters [Heart rate (HR), SDNN, RMSSD, and pNN50], frequency domain parameters (normalized low-frequency power HFn, normalized high-frequency power LFn, and the ratio of low-frequency power to high-frequency power LF/HF), and Poincaré trace parameters (SD1 and SD2) were calculated to assess the individual autonomous function.

##### Functional near-infrared spectroscopy measurement

**FIGURE 1:**
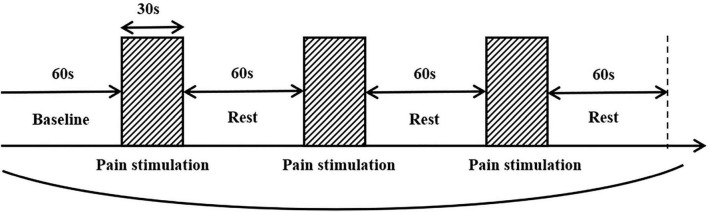
Pain stimulation paradigm.

Manual palpation, a common method of clinical pain phenotype identification, was used as a pain stimulus measure in the fNIRS measurement of cortical functional connectivity in the study. The purpose of this manipulation is to simulate the patient’s state when he or she is in the most pain. The clinician induced referred pain with the thumb and maintained a pressure of 3–4 kg/cm^2^ for 30 s (Tong et al., 2022), with 1 min interval each time, for a total of 3 times to reduce measurement error. During fNIRS monitoring, patients were instructed to sit in a relaxed position with their eyes closed throughout the procedure. The measurements began with a 1-min baseline calibration period, followed by three cycles of pain stimulation, as shown in Figure 1.

**FIGURE 2:**
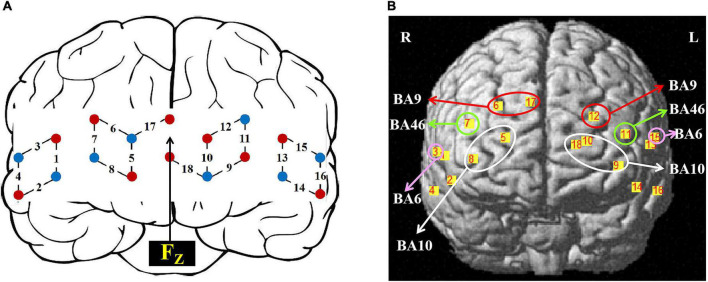
Setup of fNIRS probes and channels. **(A)** Schematic illustration of the fNIRS probe layout. **(B)** The topographical distribution of the fNIRS recording channels.

We used the Brite24 Continuous Wave system fNIRS (Artinis, Netherlands) with 18 optodes (10 light sources, 8 detectors, 10 Hz sampling rate). Two different wavelengths (752 nm and 841 nm) of NIR light were used to detect changes in oxyhemoglobin (HbO) and deoxyhemoglobin (HbR) concentrations. 18 optodes were positioned to cover the frontal cortex (30 mm distance between probes), making a total of 18 channels as shown in Figure 2A. The center of the row of the middle probe of probes was positioned approximately at FPz according to the international 10/20 system. The topographical distribution of the fNIRS channels was visualized on a standard human cortical surface using the NIRS_SPM software (Figure 2B). The channel was registered with the corresponding cortical region in the MNI space underneath and transferred to the corresponding cortical projection point on the ICBM-152 brain model (Lancaster et al., 2007). Thus, the regions of interest (ROIs) were CH5, CH8, CH9, CH10, CH18 (BA10), CH6, CH12, CH17 (BA9), CH7, CH11 (BA46), CH3, CH15 (BA6).

The fNIRS indicators include HbO, HbR, and total oxygenated concentrations, where increased HbO concentration and decreased HbR concentration are often used as indicators of activation of cortical production. In the present study, HbO and HbR were used as analytical indicators. HbO and HbR data were pre-processed in the Matlab-based NIRS_KIT toolbox, comprising a polynomial regression model for detrending, robust regression-based inverse time distribution repair for motion correction (to remove spike artifacts and baseline drift), and an infinite pulse response filter to eliminate mechanical and physiological noise. To minimize interferences such as scalp blood flow, we also used the short-distance reference channel noise regression from the toolkit. The HbO and HbR data were then analyzed using generalized linear models (GLM) to obtain statistically significant and concentration-dependent scaling factors β, reflecting the extent to which each channel is activated.

### Statistics

In this study, data from VAS, HR, and HRV before and after the music intervention were statistically analyzed using the SPSS26 software. The values were expressed as mean ± standard deviation. The Shapiro-Wilk test was used to test whether the experimentally obtained data satisfied a normal distribution. Qualitative data, such as gender, were analyzed using the χ^2^ test. Quantitative data that satisfied normal distribution, such as VAS, HR, and the indicators in HRV, were analyzed using a two-way ANOVA.

In addition, the Shapiro-Wilk test was used to check the normality of the fNIRS data. The comparison of β values in each ROI was performed with an independent samples *t*-test. For the connectivity analysis, we averaged the individual HbO and HbR concentration changes in the subjects’ ROIs. After multiple comparisons, we obtained four sets of 8 × 8 matrices as well as four brain connectivity maps. The test statistic and its corresponding *P* were given, with differences considered statistically significant at *P* < 0.05.

## Results

**FIGURE 3:**
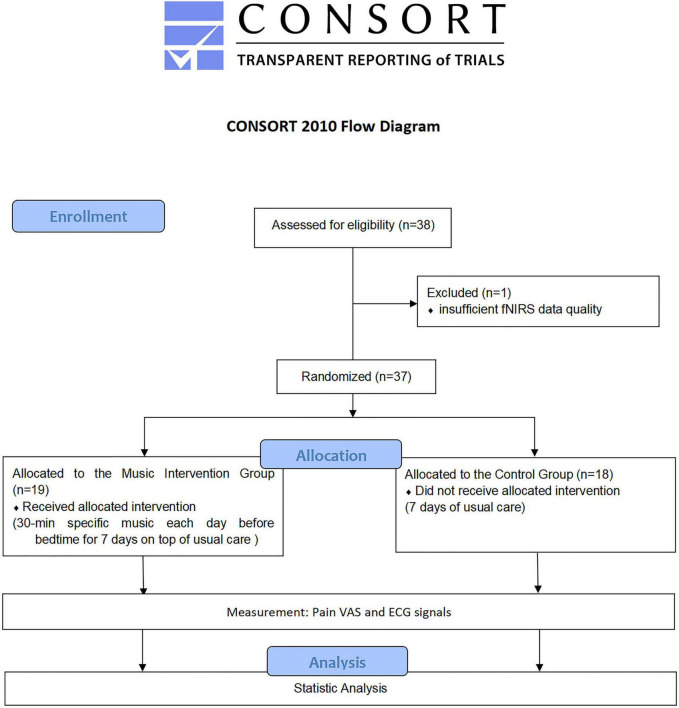
Flow chart (Boutron et al., 2017).

We recruited 38 people to participate in this study, and one person was eliminated due to insufficient fNIRS data quality (intervention: *n* = 19; control: *n* = 18) (Figure 3). The differences in general information between the two groups were not statistically significant (*P* < 0.05), as shown in Table 1.

**TABLE 1:** Comparison of general information between the two groups of patients.

Indicator	The intervention group (*n* = 19)	The control group (*n* = 18)	χ^2^ / *t*	*P*
Gender (female)	10	12	1.03	0.67
Age/years	51.00 ± 9.49	52.57 ± 11.72	–0.29	0.78

### Comparison of visual analog scale scores

As shown in Table 2, the time-by-group interaction effects were more significant (*P* < 0.01). Further simple effects test analysis yielded that in the intervention group, the VAS values were significantly higher in the pre-test than in the post-test (*F* = 31.50, *P* < 0.001, η^2^ = 0.71). In the control group, the VAS values of the pre-test and post-test were not significant (*F* = 1.00, *P* = 0.34, η^2^ = 0.07).

**TABLE 2:** Results of the analysis of the VAS of the two groups of patients in the pre-test and post-test.

	n	Pre-test	Post-tset	*F*	*P*	η^2^
The intervention group	17	7.00 ± 0.93	5.50 ± 1.69			
The control group	16	7.14 ± 0.90	6.86 ± 1.07			
Main effect of group				1.61	0.23	0.11
Main effect of time				20.83	**0.01** ^†^	0.62
Time by group interaction effects				9.63	**0.01** ^†^	0.43

^†^P < 0.01.

### Comparison of heart rate

The HR of the participants was derived based on ECG signals. As shown in Table 3, the time-by-group interaction effects were significant (*P* < 0.05). After simple effects tests, the HR was significantly higher in the pre-test than in the post-test in the intervention group (*F* = 22.39, *P* < 0.001, η^2^ = 0.67). In the control group, HR was not significant in the pre-test versus post-test (*F* = 1.14, *P* = 0.31, η^2^ = 0.09).

**TABLE 3:** Results of the analysis of HR in the two groups of patients in the pre-test and post-test.

	n	Pre-test/BPM	Post-test/BPM	*F*	*P*	η^2^
the intervention group	17	79.74 ± 5.90	75.02 ± 7.36			
the control group	16	79.13 ± 5.97	78.14 ± 6.19			
main effect of group				0.13	0.72	0.01
main effect of time				17.62	**0.01** ^†^	0.62
time by group interaction effects				7.55	**0.02** *	0.41

*P < 0.05, ^†^P < 0.01.

### Comparison of heart rate variability

Table 4 represents the results of the two-way ANOVA performed on HRV indicators. Due to significant time-by-group interaction effects (*P* < 0.05), further simple effects test analysis yielded that in the control group, the time-domain parameters (SDNN, RMSSD, pNN50), part of the frequency domain indicators (LFn, LF/HF) and the nonlinear indicator SD2 was significantly lower in the pre-test than in the post-test (*F* = 20.27, *P* < 0.05, η^2^ = 0.65; *F* = 20.27, *P* < 0.05, η^2^ = 0.65; *F* = 6.48, *P* < 0.05, η^2^ = 0.37; *F* = 11.97, *P* < 0.05, η^2^ = 0.52; *F* = 10.33, *P* < 0.05, η^2^ = 0.48; *F* = 18.39, *P* < 0.01, η^2^ = 0.63), and the frequency domain index HFn of the pre-test was significantly higher than that of the post-test (*F* = 13.63, *P* < 0.05, η^2^ = 0.55). In the intervention group, the nonlinear indicator SD1 was significantly lower in the pre-test than in the post-test (*F* = 21.69, *P* < 0.001, η^2^ = 0.66). The results demonstrate the effectiveness of the music intervention in relieving pain and reducing voluntary activity.

**TABLE 4:** HRV results of pre-test and post-test in the two groups of patients.

Indicator		The intervention group (*n* = 17)		The control group (*n* = 16)	
		
		Pre-test	Post-test	Pre-test	Post-test
Time-domain	SDNN/ms	28.34 ± 8.49	26.76 ± 9.07	22.43 ± 8.57	33.91 ± 13.29
	RMSSD/ms	24.29 ± 7.06	22.66 ± 9.69	18.11 ± 5.77	21.46 ± 6.76
	pNN50/ms	3.10 ± 3.35	2.84 ± 3.57	1.61 ± 1.11	2.55 ± 2.69
Frequency-domain	LFn	54.46 ± 24.28	53.94 ± 20.62	43.45 ± 18.86	66.49 ± 20.69
	HFn	47.00 ± 16.58	46.48 ± 12.00	58.67 ± 11.95	37.85 ± 27.38
	LF/HF	1.46 ± 0.81	1.18 ± 0.63	0.82 ± 0.51	1.82 ± 1.13
Nonlinear	SD1/ms	14.80 ± 5.36	20.02 ± 7.53	14.37 ± 2.97	16.12 ± 3.07
	SD2/ms	40.27 ± 20.70	46.24 ± 21.77	28.37 ± 12.98	44.46 ± 18.46

### Comparison of prefrontal connectivity based on changes in oxyhemoglobin/deoxyhemoglobin concentration

The activation intensity was calculated separately based on the activation level of the channels in the task, with higher β values indicating stronger activation. Table 5 shows the comparison of the activation intensity of each ROI for all subjects in the intervention and control groups, and the activation intensity in the intervention group was significantly lower than that in the control group.

**TABLE 5:** Comparison of the activation intensity of ROI brain regions between two groups of patients.

Group	n	β			
		
		BA10	BA9	BA46	BA6
The control group	17	3.99 ± 4.91	3.27 ± 4.04	2.14 ± 3.78	2.51 ± 1.84
The intervention group	16	0.60 ± 0.55	−0.10 ± 1.61	−0.64 ± 1.18	0.33 ± 1.87
*t*		2.21	2.39	2.20	2.28
*P*		**0.04** *	**0.03** *	**0.05** *	**0.04** *

*P < 0.05.

**FIGURE 4:**
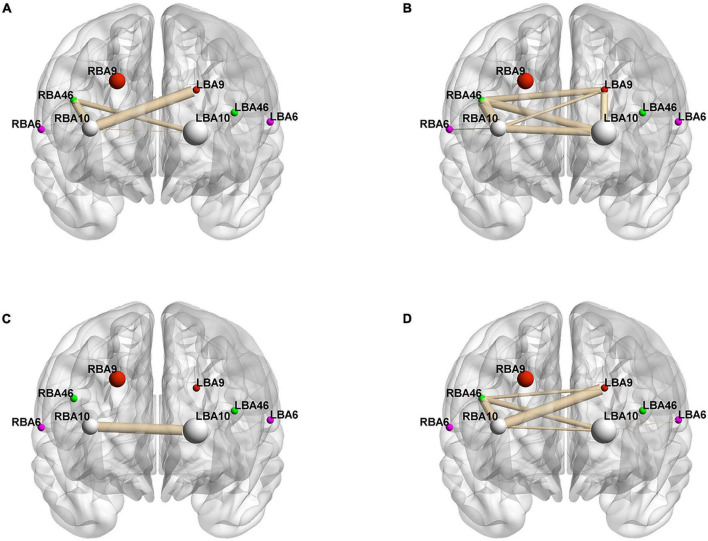
Whole-brain connectivity of ROIs in the prefrontal lobe. **(A,B)** Portray whole-brain connectivity based on changes in HbO concentration in the control and intervention groups, respectively, and **(C,D)** portray whole-brain connectivity based on changes in HbR concentration in the control and intervention groups, respectively. Where the round spheres represent each of the 8 ROIs, the beige line segments indicate the correlation between the ROIs, and the thickness indicates the degree of correlation. To make the images clearer, only significant correlations are shown in the figure (*P* < 0.05).

To better analyze the correlation between the ROIs and brain connectivity analysis, we divided the region of interest into two blocks and continued the Pearson analysis based on the experimentally measured HbO and HbR concentrations, and the results are shown in Figure 4 and Tables 6–9.

**TABLE 6:** HbO-based ROIs connectivity analysis—the control group.

	RBA9	LBA9	RBA46	LBA46	RBA6	LBA6	RBA10	LBA10
RBA9	1.00							
LBA9	0.32	1.00						
RBA46	0.64	0.67	1.00					
LBA46	0.20	–0.10	–0.32	1.00				
RBA6	–0.12	0.27	0.41	–0.33	1.00			
LBA6	–0.03	0.71	–0.01	0.19	0.05	1.00		
RBA10	0.51	**0.89** *	**0.86** *	–0.39	0.20	0.40	1.00	
LBA10	0.26	0.74	**0.83** *	–0.24	0.28	0.09	0.78	1.00

*P < 0.05.

**TABLE 7:** HbO-based ROIs connectivity analysis—the intervention group.

	RBA9	LBA9	RBA46	LBA46	RBA6	LBA6	RBA10	LBA10
RBA9	1.00							
LBA9	0.58	1.00						
RBA46	0.38	**0.90** *	1.00					
LBA46	–0.15	–0.36	–0.26	1.00				
RBA6	–0.32	0.41	0.72	0.02	1.00			
LBA6	0.03	0.13	0.09	0.27	0.16	1.00		
RBA10	0.34	**0.83** *	**0.99^†^**	–0.21	0.77	0.14	1.00	
LBA10	0.57	**0.91** *	**0.93^†^**	–0.35	0.52	0.28	**0.92^†^**	1.00

*P < 0.05, ^†^P < 0.01.

**TABLE 8:** HbR-based ROIs connectivity analysis—the control group.

	RBA9	LBA9	RBA46	LBA46	RBA6	LBA6	RBA10	LBA10
RBA9	1.00							
LBA9	0.42	1.00						
RBA46	0.41	–0.05	1.00					
LBA46	0.32	0.10	–0.26	1.00				
RBA6	–0.11	0.71	–0.07	0.10	1.00			
LBA6	0.78	0.42	–0.16	0.76	–0.01	1.00		
RBA10	0.62	0.70	0.59	0.21	0.52	0.38	1.00	
LBA10	0.55	0.76	0.38	0.38	0.70	0.47	**0.94^†^**	1.00

^†^P < 0.01.

**TABLE 9:** HbR-based ROIs connectivity analysis—the intervention group.

	RBA9	LBA9	RBA46	LBA46	RBA6	LBA6	RBA10	LBA10
RBA9	1.00							
LBA9	–0.38	1.00						
RBA46	0.05	**0.81** *	1.00					
LBA46	–0.64	–0.13	–0.60	1.00				
RBA6	–0.19	0.28	0.33	–0.16	1.00			
LBA6	–0.35	–0.13	–0.41	0.22	–0.58	1.00		
RBA10	–0.32	**0.97^†^**	**0.91** *	–0.25	0.35	–0.27	1.00	
LBA10	0.10	0.71	**0.86** *	–0.43	0.64	–0.76	**0.80** *	1.00

*P < 0.05, ^†^P < 0.01.

Figures 4A,B, and Tables 6–9 present the connectivity of prefrontal ROIs as a function of HbO concentration change. Figure 4A and Table 6 indicate significant correlations in RBA10-LBA9, RBA10-RBA46, and RBA46-LBA10 during pain in the control group (*P* < 0.05). Compared to the control group, the intervention group had significantly increased complexity of brain region connectivity, with significant correlations in LBA9-RBA46, LBA9-RBA10, and LBA9-LBA10 (*P* < 0.05) and elevated correlations in RBA10-RBA46, RBA10-LBA10 and RBA46-LBA10 (*P* < 0.01), as shown in Figure 4B and shown in Table 7.

Figures 4C,D, and Tables 8, 9 display prefrontal ROI connectivity according to HbR concentration change. As shown in Figure 4C and Table 8, there was a significant LBA10-RBA10 correlation in the control group during pain (*P* < 0.05). In the intervention group, significant correlations (*P* < 0.05) were observed in RBA10-RBA46, RBA10-LBA10, RBA46-LBA9, RBA46-LBA10, and highly significant correlations (*P* < 0.01) in RBA10-LBA9, as shown in Figure 4D and Table 9.

## Discussion

Previous studies have shown that music intervention induces rather strong vagal modulation by activating the mesencephalic and hippocampal systems of the central nervous system, promoting the secretion of endogenous opioids in the body, reducing patients’ subjective nociception, and stabilizing patients’ vital signs such as blood pressure and HR (Guerrier et al., 2021). Furthermore, music-induced analgesia is associated with the modulation of neural down-regulation of pain *in vivo*, where the prefrontal cortex is involved in extremely complex higher cognitive processes related to the perception and processing of pain and music (Koelsch et al., 2022). In this study, we compared changes in pain VAS scores, HRV, and cerebrocortical hemodynamics in patients with chronic pain following a music intervention.

In this study, VAS was used to assess patients’ subjective pain perception. As shown in Table 2, the VAS scores were lower in the intervention group, indicating that the music intervention was able to significantly reduce the patients’ subjective pain levels, providing some evidence of the positive effect of music intervention on chronic pain. This is related to the contribution of the limbic dopamine system of the brain to the affective component of pain perception (Sihvonen et al., 2022).

The HR is regulated by several reflex pathways in the brainstem and autonomic cardiac nodes, while the latter is influenced by forebrain cortical structures such as the hypothalamus, amygdala, insular cortex, and orbitofrontal cortex (Esler, 2016). Studies have shown that music can induce affective and emotional regulation through structural activity in the anteriorbrain, thereby reducing HR (Taruffi, 2021). As shown in Table 3, the HR in the intervention group was significantly lower than in the control group, indicating a significant sedative effect of the music intervention on HR. This is consistent with previous studies confirming the effectiveness of music intervention in reducing patients’ pain levels.

Heart rate variability (HRV) analysis is currently a better non-invasive tool to investigate autonomic function, reflecting the regulation of heart rhythm by the central and autonomic nervous systems. Moreover, HRV allows an objective evaluation of sympathetic and vagal nerve activity (Winbo et al., 2020). In the results of the time-domain analysis, SDNN mainly reflects the change in overall HRV activity, which is the slowly changing component of HRV. RMSSD and pNN50 primarily reflect vagal nerve activity, which reflects the rapidly evolving component of HRV. As shown in Table 4, the time-domain indices SDNN, RMSSD, and pNN50 were significantly higher in the control group during the pain period in the post-test than in the pre-test, indicating that pain stimulation led to an increase in overall autonomic and vagal activity. In addition, the post-test of time-domain parameters was lower in the intervention group than the pre-test, demonstrating that the music intervention was able to suppress the overall autonomic activity triggered by the pain stimulus, while the vagus nerve played a dominant regulatory role in the music intervention.

On the results of frequency-domain analysis, HFn reflects the functioning level of the vagus nerve, and LFn shows the regulation of the sympathetic nerve with the change in total efficiency. LF/HF reflects the balance of sympathetic and vagus nerves, with an increase, indicating active sympathetic nerves, and a decrease indicating that sympathetic and vagus nerves are imbalanced. In this study, both LFn and LF/HF were significantly increased and HFn was significantly decreased in the control group compared to the pre-test. The findings suggested that overall autonomic activity increases in response to painful stimuli, with the sympathetic nerves acting as the primary regulator. However, the music intervention inhibited the tendency, and allowed the vagus nerve to occupy an active state.

The HRV nonlinear analysis allows quantitative reflection of the structure and complexity of the RR time interval. SD1 indicates the activity of the vagus nerve. SD1 was significantly higher in the pre-test than in the post-test in the intervention group, reflecting a greater effect of the music intervention on vagal activity. SD2 reflects the change in the overall autonomic function. SD2 in the control group was significantly lower in the pre-test than in the post-test, reflecting the inhibitory effect of the music intervention on overall autonomic activity.

Previous studies have found that pain not only activates an area of the brain, but induces synchronized or coordinated areas of activity across multiple brain regions, most of which involve unilateral or bilateral BA6, BA9, BA10, and BA46 (Pascalis et al., 2015; Shi et al., 2022). It was found that the music intervention breaks the original brain connectivity network and weakens or even inactivates the connections between relevant areas of the brain, slowing the perception of pain. The prefrontal ROIs connections in the control group were lower and mostly transcerebral connections as shown in Figure 4 and Tables 5–8, consistent with the finding that pain activates the bilateral brain regions (Song et al., 2016). Brain connectivity based on HbO and HbR levels increased significantly, indicating that music intervention increased the functional coupling of pain perception and processing in the cerebral cortex (Lunde et al., 2019). Significantly enhanced connectivity of the bilateral dorsolateral prefrontal cortex and frontopolar regions represented by BA9, BA46, and BA10 would further improve the upward processing of perceptual information in patients. With the music intervention, we have seen increased connectivity in the bilateral dorsolateral prefrontal cortex, which is involved in downward maintenance and regulation of pain detection, perception, and inhibition (Lunde et al., 2019). We also found that music intervention can positively modulate the functional connections between the frontopolar area and the dorsolateral prefrontal cortex, favoring the pituitary secretion of enkephalins (Crane, 2021), thus improving functional connections in neural pathways related to pain perception and treatment, pain-reliever in patients.

Previous studies on the analgesic effects of music interventions are usually unidimensional, and there is a lack of objective evidence to verify the multidimensional effects of music interventions on patients with pain, and thus the effectiveness of music interventions to relieve pain is debatable. This study investigated synchronous changes in subjective pain scores, heart rate variability, and prefrontal cerebral hemodynamics in patients with chronic pain during music intervention. We found that the music intervention used in this experiment significantly reduced patients’ pain VAS scores, relieved HR, and significantly suppressed overall autonomic activity by innervating vagal nerve activity. This confirms the view of previous studies on the effect of music interventions on pain relief (Lee, 2016). Furthermore, we combined the collected cerebral hemodynamic signals with validated data related to functional brain activity, and the music intervention reduced HbO concentrations in brain regions associated with pain processing and increased functional coupling of pain perception and processing in the cerebral cortex. Additionally, we found that music intervention not only resulted in a significant increase in connectivity between the bilateral dorsolateral prefrontal cortex and frontopolar areas, but also positively modulated functional connectivity between frontopolar areas and the dorsolateral prefrontal cortex. By the changes in pain perception, heart rate variability, and prefrontal cerebral hemodynamic parameters in the subjects after the music intervention, we further demonstrated the effectiveness of appropriate music intervention in helping to distract and relieve anxiety and thus pain to some extent. These findings in heart rate variability and functional networks provide a physiological basis for music interventions for analgesia, contribute to understanding the pathogenesis of pain, and provide new directions for quantitative clinical assessment of pain.

However, it is difficult to fully explore the effects of various musical factors (frequency or volume) on pain patients with a single intervention approach. In the follow-up study, we will add different types of music intervention groups and design a double-blind randomized controlled trial to recruit a larger sample size of patients with chronic pain as a clinical reference. Further, we will determine the optimal music for analgesic intervention based on musical characteristics, and explore the mechanism of music intervention in improving the response of pain in brain regions with multimodal brain imaging techniques such as EEG and fMRI.

## Data availability statement

The raw data supporting the conclusions of this article will be made available by the authors, without undue reservation.

## Ethics statement

The studies involving human participants were reviewed and approved by the Medical Ethics Committee of Huadong Hospital Affiliated to Fudan University. The patients/participants provided their written informed consent to participate in this study. Written informed consent was obtained from the individual(s) for the publication of any potentially identifiable images or data included in this article.

## Author contributions

JD and PS designed and performed the experiments. JD analyzed the data and wrote the manuscript. PS, HY, and FF reviewed the manuscript. All authors approved the final manuscript.

## Abbreviations

VAS: visual analog scale
HRV: heart rate variability
fNIRS: functional near-infrared spectroscopy
HbO: oxyhemoglobin
HbR: deoxyhemoglobin
ECG: electrocardiogram
HR: heart rate.
